# Circulating Extracellular Matrix-Remodeling Biomarkers in Children with Bicuspid Aortic Valve: An Exploratory Cross-Sectional Study

**DOI:** 10.3390/diagnostics16172812

**Published:** 2026-09-01

**Authors:** Oana Iulia Man, Ximena Maria Mureșan, Mădălina Nistor, Andra Negru, Bogdan M. Tarcău, Renata Agoston, Rares Ilie Orzan, Crina Șufană, Călin Lazăr, Lucia Agoston-Coldea, Cecilia Lazea

**Affiliations:** 1 Department of Internal Medicine, Iuliu Hatieganu University of Medicine and Pharmacy, 400012 Cluj-Napoca, Romania; oana.man92@gmail.com (O.I.M.); renata.agoston@yahoo.com (R.A.); orzanrares@gmail.com (R.I.O.); luciacoldea@yahoo.com (L.A.-C.); 2 Personalized Medicine and Rare Diseases Department, Institute for Biomedical Research–MEDFUTURE, Iuliu Hatieganu University of Medicine and Pharmacy, 400349 Cluj-Napoca, Romania; ximena.muresan@medfuture.ro (X.M.M.); madalina.nistor7@gmail.com (M.N.); 3 Doctoral School of Biological and Biomedical Sciences, University of Oradea, 410087 Oradea, Romania; tarcaubogdanmihai@gmail.com; 4 1st Department of Pediatrics, Iuliu Hatieganu University of Medicine and Pharmacy, 400370 Cluj-Napoca, Romania; crina@sufana.ro (C.Ș.); calinlazar2004@yahoo.com (C.L.); cicilazearo@yahoo.com (C.L.); 5 1st Pediatrics Clinic, Emergency Pediatric Clinical Hospital, 400370 Cluj-Napoca, Romania; 6 2nd Department of Internal Medicine, Emergency County Hospital, 400012 Cluj-Napoca, Romania

**Keywords:** bicuspid aortic valve, pediatric aortopathy, extracellular matrix remodeling, matrix metalloproteinase-9, tissue inhibitor of metalloproteinase-1, transforming growth factor-β1

## Abstract

**Background/Objectives:** Bicuspid aortic valve (BAV) is frequently associated with valvular dysfunction and aortic remodeling. This exploratory study examined cross-sectional associations between circulating extracellular matrix-related biomarkers and concurrent echocardiographic characteristics in children with BAV. **Methods:** We included 43 pediatric participants—26 with BAV and 17 with tricuspid aortic valves (TAV). All participants underwent clinical assessment, transthoracic echocardiography, and serum biomarker quantification at the same study visit. MMP-2, MMP-9, TIMP-1, and TGF-β1 were measured using ELISA. Biomarker levels were compared between the BAV and TAV groups and, within the BAV group, according to the presence of concurrent aortopathy, aortic stenosis, and aortic regurgitation. **Results:** MMP-9, TIMP-1, and the MMP-9/TIMP-1 ratio were higher in the BAV group than in the TAV comparison group. Within the BAV cohort, none of the investigated biomarkers differed significantly between participants with and without aortopathy. TGF-β1 was higher in BAV patients with aortic stenosis, whereas the MMP-9/TIMP-1 ratio showed a borderline difference according to aortic regurgitation status; these subgroup findings are exploratory and limited by small sample size and multiple comparisons. **Conclusions:** In this selected pediatric cohort, circulating MMP-9 and TIMP-1 levels differed between children with BAV and the TAV comparison group. However, none of the investigated biomarkers differed significantly between BAV patients with and without aortopathy. These cross-sectional findings describe exploratory associations with the BAV phenotype but do not establish diagnostic, predictive, or prognostic utility. Larger longitudinal studies including the broader clinical spectrum of BAV and independent validation cohorts are required.

## 1. Introduction

Bicuspid aortic valve is the most frequent congenital heart disease, which can occur as an isolated malformation with a prevalence of 0.5–0.8% in the young population and 1–2% in the general population, or associated with other congenital heart disorders with a higher incidence of 50–85% [1,2]. Initial evaluation and continuous monitoring of pediatric patients is still challenging, as studies in this specific field are still scarce.

Current guidelines recommend transthoracic echocardiography for diagnosis, baseline assessment, and surveillance of BAV and the thoracic aorta [2,3]. Pediatric aortic dimensions should be interpreted using body-size-normalized Z-scores, with Z > 2 commonly used to define dilatation [3]. Freixa-Benavente et al. found good correlation between Pediatric Heart Network and Halifax Z-scores, but the Pediatric Heart Network equations produced higher values, illustrating the effect of the chosen nomogram during somatic growth [4]. Cardiovascular magnetic resonance and computed tomography can provide complementary assessment of aortic dimensions, ventricular geometry, and function, while 4D-flow imaging can characterize altered hemodynamics [1,5]. Global longitudinal strain may also identify subtle ventricular dysfunction before a reduction in ejection fraction [6].

Risk stratification and surgical treatment timing for bicuspid aortic valve-associated aortopathy are currently based mainly on maximal aortic diameter, with an ascending aortic diameter ≥60 mm or ≥4.25 cm/m^2^ considered associated with a higher risk of an adverse event. However, there are several drawbacks of this method, as >90% of life-threatening aortic complications occur with an aortic diameter <50–55 mm [7].

Aortic enlargement and aneurysm development, mostly of the ascending segment, are elements of the complex called bicuspid-aortopathy, which can occur in the absence of valvular dysfunction (stenosis or regurgitation). It is considered as “Marfan syndrome-like” by some authors because of its more malignant behavior compared to other causes of aortic aneurysm formation. Although the mechanisms underlying BAV and TAV aneurysms were thought to be very similar, several researchers have addressed the management of BAV-associated aortopathy, revealing a specific pathogenetic signature [2]. The embryological development of cardiac structures and adaptive remodeling of the aortic wall are essential for understanding the physiopathogenesis of congenital aortopathies [8]. Genetic and hemodynamic factors are intertwined in the development and progression of bicuspid aortic valves and associated disorders, contributing to aortic wall weakening and subsequent valvular alterations. The genetic theory is supported by high heritability, as evidenced by familial clustering, with aortopathy observed in first-degree relatives of patients with BAV, even though most cases are sporadic [1,9]. Multiple inheritance models have been described, including autosomal dominant with low penetrance, variable expressivity, and male predominance, X-linked, and familial modes [1,10]. Several gene mutations have been identified, such as *NOTCH1*, *GATA4*, *GATA5*, *GATA6*, *ROBO4*, *MAT2A*, *ADAMTS19*, *TBX20*, *NKX2-5* [1,10]. On the other hand, hemodynamic theory is supported by extensive evidence that mechanical causes and blood flow disturbances drive secondary changes in the biomechanical properties and composition of the aortic wall, leading to progressive weakening, aneurysm formation, and eventually aortic dissection or rupture [11].

Medial cystic necrosis or degeneration is the main alteration of the aortic wall structure in bicuspid aortopathy, characterized by the development of smooth muscle cells within the aortic tunica media that are detached from the extracellular matrix and stimulate the release of matrix metalloproteinases and their tissue inhibitors [9]. Shipulina et al. [12] investigated histopathological patterns in aortic aneurysms associated with BAV and TAV, showing elastic fiber fragmentation in BAV, compared to translamellar mucoid accumulation, inflammatory and atherosclerotic changes in TAV. In addition, in contrast with TAV aortopathy, microscopic BAV aortopathy reveals normal fiber architecture, loss of smooth muscle cells with apoptosis, medial degeneration, and lower fibrillin content [13].

Together with multimodality imaging, circulating biomarkers have been extensively studied as molecular underpinnings and potential indicators of extracellular matrix remodeling in BAV-associated disease. However, evidence regarding their clinical utility remains limited, particularly in pediatric populations, and cross-sectional biomarker differences cannot establish prediction or prognosis [7,8].

Most studies have focused on the balance between matrix metalloproteinases (MMPs) and their tissue inhibitors (TIMPs). MMP-2 and MMP-9 contribute to extracellular matrix turnover, and differences in their circulating concentrations have been reported between BAV and TAV populations [13].

TGF-β1 signaling pathway plays a crucial role in the development of aortic dilation, being a cornerstone in the regulation of many physiological homeostases, including maintenance of the normal structure and function of the aorta [14]. In BAV patients, TGF-β1 signaling, which is responsible for the development of the intimal layer of the aorta, is impaired, leading to a lack of intimal thickening after birth and throughout the lifespan. Also, dysregulation of TGF-β1 signaling disrupts the balance between matrix deposition and degradation, resulting in pathological remodeling of the extracellular matrix and alteration of the structural integrity of the vascular wall [15].

Recent pediatric studies highlight the heterogeneity of BAV-associated aortic remodeling. In a cross-sectional study of 73 children with BAV, Făgărășan et al. found that, among MMP-1, MMP-2, MMP-9, and TIMP-1, only TIMP-1 differed between children with and without aortic dilatation, with higher levels in those without dilatation [16]. Kalay Şentürk et al. reported higher MMP-2, MMP-9, and MMP-2/TIMP-1 ratio levels in 40 children with BAV than in 40 healthy controls, whereas associations were less consistent when analyses were restricted to the BAV group [17]. In the multicenter MIBAVA cohort, ascending aortic dilatation was not only associated with valve morphology, aortic stenosis, aortic regurgitation, and older age but was also present without valvular dysfunction, supporting a multifactorial model [18].

The four biomarkers were selected as a focused, hypothesis-driven panel representing complementary components of extracellular matrix remodeling. MMP-2 and MMP-9 are gelatinases involved in matrix proteolysis; TIMP-1 regulates MMP activity and provides information on the protease-inhibitor balance; and TGF-β1 represents a profibrotic signaling pathway implicated in aortic wall remodeling. The panel was not intended as comprehensive or hypothesis-free biomarker discovery; other MMPs, TIMPs, inflammatory mediators, and emerging matrix markers were outside the scope of this study. In addition, marker selection was influenced by concerns about assay availability and pediatric sample volume constraints.

We hypothesized that circulating biomarkers related to extracellular matrix remodeling may differ between pediatric patients with BAV and TAV and may show cross-sectional associations with concurrent echocardiographic characteristics. Accordingly, we explored differences in MMP-2, MMP-9, TIMP-1, and acid-activated TGF-β1 concentrations between groups and within the BAV cohort according to aortopathy and valvular dysfunction status.

## 2. Materials and Methods

### 2.1. Study Design and Patients’ Characteristics

We conducted a single-center exploratory observational study with cross-sectional clinical, echocardiographic, and biomarker assessment. Biomarker sampling and echocardiography were performed at the same visit, without longitudinal follow-up; the design therefore assessed concurrent associations and was not intended to evaluate prediction, diagnostic performance, or prognosis. Pediatric participants evaluated at the Pediatric Cardiology Department of the 1st Pediatric Clinic, Emergency Pediatric Clinical Hospital, Cluj-Napoca, Romania, between January 2023 and January 2026 were screened using prespecified eligibility criteria and consecutive sampling. Of 105 screened patients, 62 were excluded: age or other eligibility criteria (*n* = 9), suboptimal echocardiographic image quality (*n* = 7), additional congenital heart disease or arrhythmia (*n* = 22), prior intervention, syndromic aortopathy, or relevant chronic disease (*n* = 12), and declined participation or incomplete study procedures (*n* = 12). The final cohort comprised 43 participants: 26 with BAV and 17 with TAV. The TAV participants constituted a contemporaneous clinical comparison group, not healthy controls, and no individual matching was performed.

Eligibility criteria were applied to both groups. Inclusion required age <18 years, adequate transthoracic echocardiographic image quality, appropriate visualization of aortic valve and aortic morphology, and consent to all study procedures. Exclusion criteria included suboptimal acoustic windows, additional major congenital heart disease or arrhythmia, previous cardiac intervention, syndromic aortopathy or another genetic syndrome, known chronic systemic disorders expected to materially affect the biomarkers, systemic inflammatory disease, and current systemic corticosteroid treatment at enrollment or blood sampling. The TAV group was defined by tricuspid aortic valve morphology and served as a clinical comparison group. One TAV participant had mild aortic regurgitation without hemodynamic impact; two TAV participants were receiving a beta-blocker or an angiotensin-converting enzyme inhibitor for primary hypertension. Eligibility was evaluated by medical history, review of available records, clinical examination, echocardiography, and the study laboratory assessment. No dedicated laboratory screen for occult systemic disease was undertaken in otherwise asymptomatic participants.

Participants were categorized by age as infants (0–23 months), children (24–144 months), or adolescents (145–215 months), consistent with the age categories used in the descriptive tables. Within the BAV group, concurrent aortic dilatation, aortic stenosis, and aortic regurgitation were classified according to current nomenclature and guideline-based criteria [19]. Data were recorded on standardized datasheets in accordance with privacy requirements.

**Figure 1:**
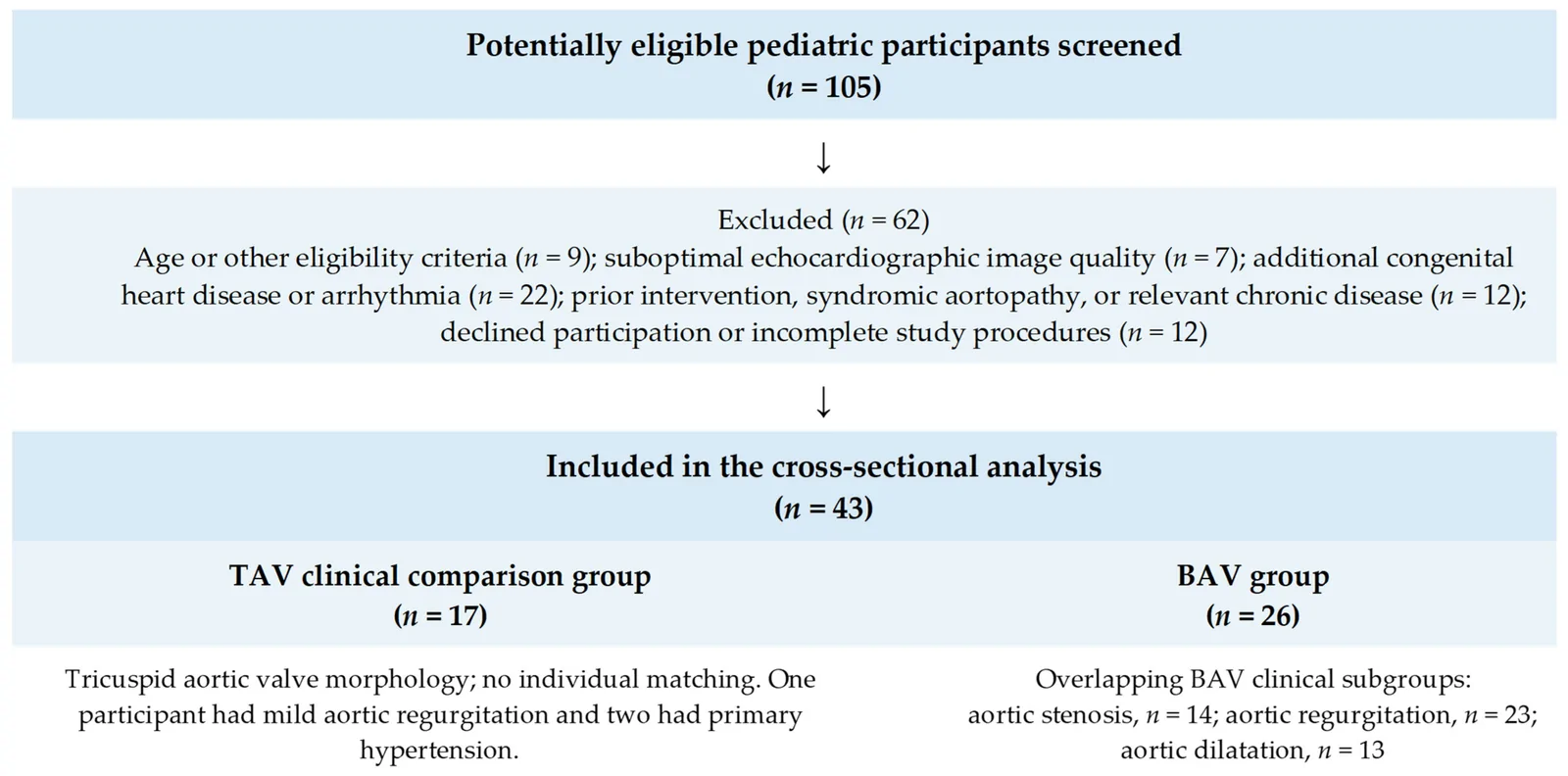
Participant flow. BAV clinical subgroups overlap and are not mutually exclusive. Abbreviations: TAV, tricuspid aortic valve; BAV, bicuspid aortic valve.

Participant recruitment and analysis are summarized in Figure 1.

### 2.2. Clinical Evaluation and Data Collection Protocol

All patients included in the study were evaluated according to a standard protocol encompassing medical history, physical examination, 12-lead electrocardiogram, transthoracic echocardiography, and blood analysis.

At the beginning, each of the included patients in the present study was interviewed for demographic and clinical data comprising gender, geographical area, age at diagnosis of BAV disease, and age at inclusion in the study, specific signs or symptoms at diagnosis (cardiac murmur, chest pain, fatigue, syncope, dyspnea, palpitations), personal medical and familial history of BAV disease, cardiac medication (beta-blockers). Additional information was obtained from electronic medical records and through telephone interviews.

Furthermore, a complete and thorough clinical examination was conducted, and relevant data were documented. Height and weight were measured by trained professionals, and body mass index and body surface area were calculated using specific mathematical formulas and online resources [20]. Heart rate and systolic and diastolic blood pressure were also measured by the auscultatory method in accordance with current European and American recommendations for the measurement, definition, and classification of pediatric hypertension, after 3–5 min of sitting in a quiet room, using an appropriate cuff size covering 80–100% of the individual’s arm circumference, with at least 40% width, and the average values of three repeated measurements in both arms with an interval of 3 min between them were then recorded [21,22].

Electrocardiography was then performed to comprehensively assess cardiac status and to identify any possible abnormalities.

### 2.3. Echocardiography Assessment

Transthoracic echocardiography was performed by two experienced pediatric cardiologists using a Vivid S7 system (General Electric, Wauwatosa, WI, USA) with an X-51 transducer. Measurements were obtained according to current recommendations [2,3,23,24,25]. For the sinuses of Valsalva, sinotubular junction, and ascending aorta, Z-scores were calculated using the Halifax equations reported by Warren et al. [26], with body surface area calculated using the Boyd formula and the systolic inner-edge-to-inner-edge convention. The same reference system was used for all participants. Missing measurements were analyzed as available cases; no values were imputed, and measurement-specific denominators are reported in the Results Section tables.

The aortic valve was evaluated in the parasternal short-axis view to describe morphology and function. The transverse annular diameter was measured in diastole at the leaflet hinge points using the inner-edge-to-inner-edge technique. Because this annular measurement did not follow the systolic convention of the Halifax equations, it is reported only as a raw diameter and was not included in Z-score analyses or in the definition of aortopathy. Aortic stenosis was evaluated by continuous-wave Doppler from multiple views, using the simplified Bernoulli equation; severity was classified from the maximal peak gradient as mild (25–50 mmHg), moderate (>50 to <70–80 mmHg), or severe (>70–80 mmHg). Aortic regurgitation severity was assessed by an integrated qualitative, semiquantitative, and quantitative approach, including jet characteristics, vena contracta, pressure half-time, descending-aortic flow reversal, and left-ventricular size.

Maximal diameters of the sinuses of Valsalva, sinotubular junction, and tubular ascending aorta were obtained from the parasternal long-axis view in mid-systole using the inner-edge-to-inner-edge technique, consistent with the Halifax reference convention [26]. Raw diameters of the aortic arch and descending thoracic aorta were also recorded when available.

Left ventricle (LV) systolic function was approached by determining the fractional shortening (FS) using the standard M-mode method and the ejection fraction (EF) through the 2D biplane Simpson method, which calculates a volume based on diastolic and systolic area measurements of the LV from the apical 4- and 2-chamber views.

The original sampling frame focused on children with clinically manifest typical BAV valvulo-aortopathy attending a tertiary pediatric cardiology service. Complex valvulo-aortopathy, previous intervention, syndromic aortopathy, additional major congenital heart disease, and uncomplicated BAV were outside the sampling frame. This restriction enriched the BAV cohort for established valvular or aortic abnormalities and limits generalizability to the full pediatric BAV spectrum. BAV morphology was classified as fused BAV (1RL, 1RN, or 1LN), two-sinus BAV (2LL or 2AP), or partial-fusion BAV (type 3) [19]. Aortic dilatation was defined as a Halifax Z-score >2 at the sinuses of Valsalva, sinotubular junction, or ascending aorta. Root, ascending, and extended phenotypes were defined by the involved aortic segments.

### 2.4. Blood Sampling and Laboratory Testing

Two peripheral venous blood samples were obtained after an 8 h overnight fast on the same day as clinical assessment and echocardiography. Tubes were centrifuged at 4000 rpm for 10 min; serum was aliquoted into Eppendorf vials and stored at −80 °C until analysis. Each analyzed aliquot underwent one freeze–thaw cycle.

Biochemical analysis of the following routine tests was carried out in the Clinical Laboratory of the Emergency Pediatric Clinical Hospital from Cluj-Napoca, Romania: troponin, N-terminal pro-B-type natriuretic peptide (NT-proBNP), creatine kinase (CK), total cholesterol, high-density lipoprotein cholesterol (HDL-cholesterol), low-density lipoprotein cholesterol (LDL-cholesterol), triglycerides.

Serum TIMP-1, MMP-2, MMP-9, and TGF-β1 concentrations were measured in the Department of Translational Medicine, Institute for Biomedical Research–MEDFUTURE, Cluj-Napoca, Romania, using DuoSet sandwich ELISA kits (R&D Systems, Minneapolis, MN, USA): TIMP-1, DY970-05 (31.3–2000 pg/mL); MMP-2, DY902 (0.625–20 ng/mL); MMP-9, DY911-05 (31.3–2000 pg/mL); and TGF-β1, DY240-05 (31.3–2000 pg/mL). Four candidate dilutions were tested for each analyte in six randomly selected samples with the calibration curve; final dilution factors were 1:50 for MMP-2, 1:200 for MMP-9 and TGF-β1, and 1:500 for TIMP-1. TGF-β1 samples were acid-activated for 10 min with 1 N HCl and neutralized with 1.2 N NaOH/0.5 M HEPES; results therefore represent acid-activated total immunoreactive TGF-β1, not biologically active circulating TGF-β1. Samples were pseudonymized, randomized across plates, and assayed in duplicate by personnel blinded to valve phenotype and echocardiographic classification. The same standard and four bridge samples were included on each plate to monitor inter-plate consistency. Absorbance was read using a CLARIOstar instrument (BMG LABTECH, Ortenberg, Germany), and 4-parameter logistic calibration was performed in MARS software. Duplicate values were averaged, concentrations were corrected for dilution, and final values are reported in ng/mL. Prespecified run-level intra-assay/inter-assay coefficient-of-variation thresholds and a formal rule for values outside the calibration range were not available in the source assay documentation and are therefore not reported retrospectively.

### 2.5. Statistical Analysis

Data collection was conducted with the help of Microsoft Office Excel, documenting all variables of interest during the recruitment and enrolment process.

The distribution of quantitative variables was assessed using Kolmogorov–Smirnov or Shapiro–Wilk tests and graphical inspection. Categorical variables are presented as *n* (%). Continuous variables are presented as mean ± standard deviation for approximately normally distributed data or median (25–75th percentile) otherwise.

Analyses were performed using R version 4.6.0. No formal a priori sample-size calculation was performed; all consecutively eligible participants recruited during the predefined period were included. Between-group comparisons used Welch’s *t*-test or the Mann–Whitney U test for continuous variables and Fisher’s exact or chi-square tests for categorical variables, as appropriate. Focused associations between biomarker concentrations and aortic Z-scores were explored using Spearman’s rank correlation coefficient. Coefficients are reported with direction, magnitude, nominal two-sided *p*-values, and available-case *n*; no qualitative strength categories were applied because such cutoffs are arbitrary and context dependent [27]. No prespecified confirmatory primary endpoint or multivariable regression model was used. In view of the small sample and the number of comparisons, all analyses are exploratory; no formal multiplicity correction was applied, and nominal *p*-values should not be interpreted as confirmatory. Descriptive effect estimates and 95% confidence intervals are reported where supported by the available analysis output.

### 2.6. Ethical Considerations

The study protocol was approved by The Ethics Committee of Iuliu Hațieganu University of Medicine and Pharmacy from Cluj-Napoca, Romania (approval no. 250/30.06.2021), as well as by The Institutional Ethics Committee of The 1st Pediatrics Clinic, Emergency Pediatric Clinical Hospital from Cluj-Napoca, Romania (approval no. 92/09.01.2023). The entire research was conducted in accordance with the Declaration of Helsinki. At the time of enrollment in the study, pediatric patients assented to participation after being provided with all the details about clinical evaluation, cardiac ultrasound assessment, and blood withdrawal for biomarker quantification, using images and/or vocabulary adapted to their educational level and age category (5 to 10 years, 11 to 14 years, and 15 to 18 years, respectively). Moreover, at least one legal representative for each patient signed a written informed consent prior to inclusion in the study.

## 3. Results

### 3.1. Baseline Features of the Study Groups

The study population comprised 43 pediatric participants, classified as 26 with BAV and 17 with TAV according to aortic valve morphology. The median age was 110 months in the BAV group and 163 months in the TAV group. Male participants represented 84.6% and 58.8% of the respective groups. These differences indicate residual age and sex imbalance; therefore, the groups are not described as individually matched.

As for clinical data, BMI and BSA distributions were broadly similar between groups, although residual confounding by age, sex, body size, and somatic growth cannot be excluded. Among BAV participants, 23 (88.5%) had a cardiac murmur at diagnosis, three (11.5%) were receiving a beta-blocker, and none reported a family history of BAV. As previously mentioned, in the TAV group, one patient had mild aortic regurgitation without hemodynamic impact and without any other cardiovascular abnormalities; two patients had a beta-blocker or an angiotensin-converting enzyme inhibitor in their medication regimens, indicated for primary hypertension.

In terms of biochemical analysis, routine laboratory tests revealed higher NT-proBNP in BAV pediatric patients (median 88.5 pg/mL) than in the TAV comparison group (median 27.02 pg/mL) (*p* = 0.03), and higher CK (117 vs. 78 u/L, *p* = 0.13). No significant differences were found between circulating lipids.

Baseline features of the study groups are summarized in Table 1.

### 3.2. Echocardiography Characteristics of the Study Groups

Aortic stenosis was present in 14 of 26 BAV participants (53.8%) and in no TAV participant. Aortic regurgitation was reported in 23 of 26 BAV participants (88.5%) and one of 17 TAV participants (5.9%), while aortic dilatation was present in 13 of 26 BAV participants (50.0%) and no TAV participant. The ascending-aortic Z-score was higher in the BAV group (median 1.79) than in the TAV comparison group (median −0.18). These cross-sectional differences characterize the selected cohort and should not be interpreted as prospective risk estimates.

The echocardiography characteristics of the study groups are summarized in Table 2.

The 26 BAV participants comprised 6 with right–left cusp fusion (1RL), 7 with right-non-coronary cusp fusion (1RN), 2 with left-non-coronary cusp fusion (1LN), and 11 with partial-fusion BAV (type 3); no two-sinus BAV phenotype was recorded. Aortic dilatation was present in 4/6 participants with 1RL, 2/7 with 1RN, 0/2 with 1LN, and 7/11 with partial-fusion BAV. Owing to the small phenotype subgroups, these distributions are descriptive.

The results described above are synthesized in Table 3.

### 3.3. Serum Biomarkers Quantified by ELISA

In unadjusted BAV-versus-TAV comparisons, MMP-9 was 346.05 (229.80–534.95) versus 152.10 (88.56–213.15) ng/mL (nominal *p* < 0.05; difference between reported group medians, 193.95 ng/mL); TIMP-1 was 168.25 (152.32–181.72) versus 132.20 (91.48–165.60) ng/mL (*p* = 0.02; median difference, 36.05 ng/mL); and the MMP-9/TIMP-1 ratio was 1.98 (1.35–3.58) versus 1.30 (1.02–1.87) (*p* = 0.01; median difference, 0.68). MMP-2 (195.44 [155.24–199.80] vs. 168.30 [154.60–196.40] ng/mL; *p* = 0.21) and TGF-β1 (105.40 [94.72–117.05] vs. 86.22 [68.09–114.60] ng/mL; *p* = 0.12) did not differ significantly. These descriptive, unadjusted results characterize concurrent group differences and do not establish biomarker specificity or diagnostic performance.

**Figure 2:**
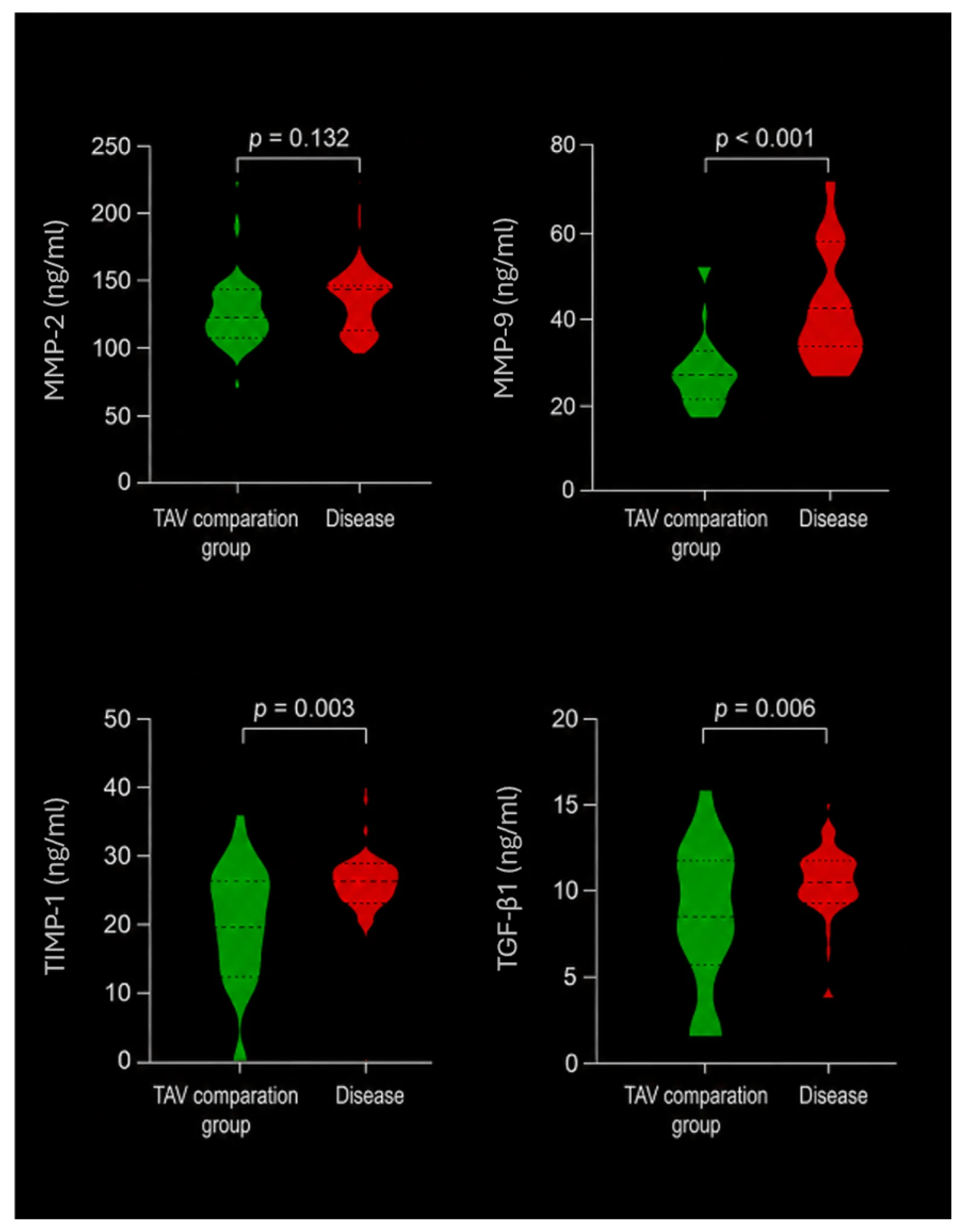
Exploratory distributions of serum extracellular matrix-related biomarkers by valve phenotype. In the original plot labels, “Disease” denotes the BAV group and versus TAV clinical comparison group. The dashed lines correspond to the median, the 25th and the 75th percentiles for each biomarker serum concentration. Abbreviations: MMP-2, matrix metalloproteinase-2; MMP-9, matrix metalloproteinase-9; TAV: tricuspid aortic valve; TIMP-1, tissue inhibitor of metalloproteinase-1; TGF-β1, transforming growth factor-β1.

Figure 2 presents the exploratory distributions of the four biomarkers according to valve phenotype. Substantial overlap between groups is evident, and neither a diagnostic threshold nor classification performance was evaluated.

An unadjusted whole-cohort comparison suggested higher MMP-9 concentrations among participants classified as having aortopathy. Because aortopathy status was strongly confounded with BAV status, this comparison is not interpreted as an aortopathy-specific association. The clinically relevant exploratory comparisons were therefore restricted to the BAV cohort.

Within the BAV cohort, none of the investigated biomarkers differed significantly between participants with and without aortopathy (Table 4). MMP-9 and the MMP-9/TIMP-1 ratio were numerically lower in participants with aortopathy. These findings do not support an aortopathy-specific biomarker conclusion in the present sample. As expected from the subgroup definition, aortic Z-scores at the sinuses of Valsalva and ascending aorta were higher in the aortopathy group.

Whole-cohort biomarker comparisons according to aortic stenosis were not interpreted because stenosis status was strongly confounded with BAV phenotype.

Within the BAV group, TGF-β1 was higher among participants with aortic stenosis at the nominal *p* = 0.03 level. MMP-2 and MMP-9 also had nominal *p* = 0.07, whereas the remaining biomarkers did not differ significantly. These subgroup results are exploratory, were not adjusted for multiple comparisons, and require external validation (Table 5).

Whole-cohort biomarker comparisons according to aortic regurgitation were likewise not interpreted because regurgitation status was strongly confounded with BAV phenotype. Within the BAV cohort, the MMP-9/TIMP-1 ratio was higher in participants with aortic regurgitation at a borderline nominal *p*-value of 0.05. This comparison is highly uncertain because only three BAV participants did not have regurgitation (Table 6).

### 3.4. Associations Between Echocardiographic Measurements and Circulating Biomarker Levels

Focused exploratory correlations between biomarker concentrations and body-size-normalized Z-scores at the sinuses of Valsalva, sinotubular junction, and ascending aorta are presented separately for BAV and TAV participants in Table 7 and Table 8. Available-case denominators are reported for every estimate. Annular Z-score correlations were removed because the annulus had been measured in diastole rather than according to the Halifax systolic convention.

Within the BAV group, none of the focused biomarker–aortic Z-score correlations reached the nominal *p* < 0.05 level. The sinotubular-junction estimates were based on only 12 available observations and remain particularly imprecise.

Within the TAV comparison group, TIMP-1 was inversely correlated with ascending-aortic Z-score (ρ = −0.54; nominal *p* = 0.02). This isolated nominal association should be interpreted cautiously in view of multiple exploratory testing; no sinotubular-junction correlation was calculated from the single available observation.

## 4. Discussion

This exploratory cross-sectional study identified unadjusted differences in selected extracellular matrix-related biomarkers between a clinically enriched pediatric BAV group and a TAV clinical comparison group. MMP-9 and TIMP-1 concentrations were higher in BAV, but contemporaneous sampling and echocardiography establish association only; they cannot establish temporal sequence, early detection, prediction, prognosis, or risk stratification.

The BAV cohort had frequent valvular dysfunction and aortic dilatation, reflecting tertiary-care recruitment and the exclusion of uncomplicated BAV. The findings therefore describe a selected clinical spectrum and should not be interpreted as population prevalence or risk estimates. Residual age, sex, body-size, hypertension, and medication differences further limit attribution of the unadjusted biomarker differences to BAV alone.

The MIBAVA study provides relevant pediatric context: among 2122 patients with BAV, 50% had ascending aortic dilatation; right-noncoronary cusp fusion, greater aortic stenosis or regurgitation, and older age were associated with dilatation, yet dilatation was also present in 37% of patients without stenosis or regurgitation [18]. In our much smaller cohort, BAV morphology, valvular dysfunction, and aortopathy were strongly interrelated and their independent effects could not be disentangled.

The most important distinction is between biomarkers associated with the BAV-versus-TAV group comparison and biomarkers specifically associated with aortopathy within BAV. None of the four biomarkers differed significantly between BAV participants with and without aortopathy. This is consistent with the need for caution raised by recent pediatric studies: Făgărășan et al. reported higher TIMP-1 in BAV participants without aortic dilatation [16], while Kalay Şentürk et al. found stronger biomarker differences in the combined BAV-versus-control comparison than in analyses restricted to BAV [17].

Focused correlations with aortic Z-scores were limited by missing data and small available-case samples. No focused correlation reached nominal significance in BAV; the isolated inverse TIMP-1–ascending-aorta association in TAV requires replication and should not be interpreted mechanistically or prognostically.

The nominal association of TGF-β1 with aortic stenosis and the borderline MMP-9/TIMP-1 difference by regurgitation status are hypothesis-generating only because of multiple testing and sparse subgroups. The between-group differences in MMP-9 and TIMP-1 appear to be associated primarily with the BAV phenotype and should not be interpreted as specific markers of BAV-related aortopathy. This distinction is supported by the absence of significant biomarker differences between BAV patients with and without aortopathy. Prior pediatric and adult studies have reported heterogeneous associations among MMPs, TIMPs, TGF-β1, aortic dimensions, and aortic elastic properties [17,28,29], which may reflect differences in phenotype, age, assay procedures, imaging definitions, and confounding.

Diagnostic or prognostic performance was not assessed. ROC curves, AUC, sensitivity, specificity, predictive values, cutoffs, and incremental value beyond echocardiography were not calculated. Post hoc ROC analysis in this small, selected cohort would be unstable and prone to optimism; diagnostic-performance evaluation should therefore await a larger independent cohort.

Strengths include combined clinical, echocardiographic, and laboratory assessment in an underexplored pediatric population. This study has several important limitations. Its cross-sectional design does not establish temporal relationships, prediction, or prognosis. Recruitment from a tertiary pediatric cardiology center and exclusion of uncomplicated BAV introduced selection and spectrum bias and limit generalizability to the broader pediatric BAV population. BAV status was strongly confounded with aortopathy and valvular dysfunction in whole-cohort comparisons. The small sample size, sparse clinical subgroups, multiple exploratory comparisons, and missing echocardiographic measurements increased statistical uncertainty and the risks of type I and type II error. Residual confounding by age, sex, BSA, and somatic growth cannot be excluded. Finally, the study lacked longitudinal follow-up and an independent validation cohort; therefore, no conclusions regarding diagnostic, predictive, or prognostic utility can be drawn.

Future multicenter studies should recruit the full pediatric BAV spectrum, including uncomplicated BAV, use standardized imaging and laboratory protocols, and follow biomarker trajectories longitudinally. Candidate findings should be prespecified and validated independently before diagnostic or prognostic performance is considered.

## 5. Conclusions

In this selected pediatric cohort, circulating MMP-9 and TIMP-1 concentrations differed in unadjusted comparisons between the BAV and TAV clinical comparison groups. However, none of the investigated biomarkers differed significantly between BAV participants with and without aortopathy. These cross-sectional findings are exploratory and do not establish a biomarker signature specific to BAV-related aortopathy or any diagnostic, predictive, or prognostic utility. Larger longitudinal studies including uncomplicated BAV and independent validation cohorts are required.

## Figures and Tables

**Table 1:** Baseline characteristics of the study population.

Parameter		BAV Group (*n* = 26)	TAV Group (*n* = 17)	*p*-Value
Demographic features				
Age at inclusion, months		110 (62.25–173.75)	163 (129.00–175.00)	0.05
Age group	Infant	4 (15.4)	1 (5.9)	0.26
	Child	12 (46.1)	5 (29.4)	
	Adolescent	10 (38.5)	11 (64.7)	
Gender	Male	22 (84.6)	10 (58.8)	0.08 OR = 0.26 (CI 0.04–1.34)
	Female	4 (15.4)	7 (41.2)	
Area	Urban	18 (69.2)	10 (58.8)	0.52 OR = 0.64 (CI 0.14–2.76)
	Rural	8 (30.8)	7 (41.2)	
Clinical data				
Body mass index, kg/m^2^		17.08 (15.21–18.26)	17.62 (15.57–21.25)	0.51
Body surface area, m^2^		1.08 (0.77–1.49)	1.32 (1.13–1.54)	0.19
Heart rate, bpm		87 (75–107)	80 (73–89)	0.17
Systolic blood pressure, mmHg		100 (93.75–110)	110 (100–115.25)	0.06
Diastolic blood pressure, mmHg		65 (60–70)	67.5 (62–76.25)	0.27
Cardiac murmur at diagnosis, yes		23 (88.5)	N/A	N/A
Family history of BAV, yes		0 (0)	0 (0)	N/A
Cardiac medication, yes		3 (11.5)	2 (11.8)	1
Routine laboratory tests				
NT-proBNP, pg/mL		88.5 (49.95–130)	27.02 (22.45–71.40)	0.03
CK, U/L		117 (80–148)	78 (56.7–80.6)	0.13
Total cholesterol, mg/dL		167 (155–180)	160.5 (139.97–178.47)	0.59
HDL cholesterol, mg/dL		56 (48.9–64.4)	59.5 (54.62–63.67)	0.54
LDL cholesterol, mg/dL		95.5 (80.2–112.4)	89.9 (76.44–109.14)	0.60
Triglycerides, mg/dL		60.5 (42.5–73.6)	50.3 (45.1–73)	0.88

Continuous data are presented as mean ± SD or median (25–75th percentile), as appropriate; categorical data are *n* (%). Comparisons used Welch’s *t*-test or the Mann–Whitney U test for continuous variables and the chi-square or Fisher’s exact test for categorical variables, as appropriate. Abbreviations: BAV, bicuspid aortic valve; TAV, tricuspid aortic valve; NT-proBNP, N-terminal pro-B-type natriuretic peptide; CK, creatine kinase; HDL, high-density lipoprotein; LDL, low-density lipoprotein; OR, odds ratio; CI, confidence interval; N/A, not applicable.

**Table 2:** Transthoracic cardiac ultrasonography features of study patients.

Parameter		BAV Group (*n* = 26)	TAV Group (*n* = 17)	*p*-Value
Aortic stenosis, yes		14 (53.8)	0 (0)	N/A
Aortic regurgitation, yes		23 (88.5)	1 (5.9)	<0.001
Aortic dilatation, yes		13 (50)	0 (0)	N/A
Annulus Ao	mm	17 (15–20.25)	18 (16–19)	0.58
Ao SV	mm	23 (20–26)	22.5 (21–26)	0.93
	Z score	0.78 (0.23–1.34)	0.44 (−0.39–0.86)	0.30
Ao STJ	mm	20 (15.87–23.75)	22 (22–22)	N/A
	Z score	1.28 (0.88–2.01)	1.04 (1.04–1.04)	N/A
Asc Ao	mm	20.75 (18.12–27.37)	20 (19–23)	0.33
	Z score	1.79 (0.70–2.78)	−0.18 (−0.85–0.21)	<0.05
LVEDV, mL		38 (32.75–42)	41 (39–46)	0.09
LVESV, mL		24 (20–26.75)	27 (25–30)	0.04
EF, %		66.03 ± 6.23	65.70 ± 4.71	0.85
FS, %		37 (33–40)	36 (33–37)	0.36

Continuous data are presented as mean ± SD or median (25–75th percentile), as appropriate; categorical data are *n* (%). Comparisons used Welch’s *t*-test or the Mann–Whitney U test for continuous variables and the chi-square or Fisher’s exact test for categorical variables, as appropriate. Abbreviations: BAV, bicuspid aortic valve; TAV, tricuspid aortic valve; Annulus Ao, aortic annulus; Ao SV, sinuses of Valsalva; Ao STJ, sinotubular junction; Asc Ao, ascending aorta; LVEDV, left-ventricular end-diastolic volume; LVESV, left-ventricular end-systolic volume; EF, ejection fraction; FS, fractional shortening; N/A, not applicable. No inferential comparison was performed for Ao STJ because only one TAV observation was available.

**Table 3:** Aortic valve and aortic morphology distribution within the BAV group.

Parameter		BAV Phenotype						
		1RL (*n* = 6)	1RN (*n* = 7)	1LN (*n* = 2)	2AP (*n* = 0)	2LL (*n* = 0)	3 (*n* = 11)	*p*-Value
Aortic dilatation								
Yes		4 (66.7)	2 (28.6)	0 (0)	N/A	N/A	7 (63.6)	N/A
No		2 (33.3)	5 (71.4)	2 (100)	N/A	N/A	4 (36.4)	
Phenotype	Root	1 (16.7)	0 (0)	0 (0)	N/A	N/A	1 (9.1)	N/A
	Ascending	3 (50)	1 (14.3)	0 (0)	N/A	N/A	4 (36.4)	
	Extended	0 (0)	1 (14.3)	0 (0)	N/A	N/A	2 (18.2)	
Aortic stenosis								
Yes		2 (33.3)	5 (71.4)	1 (50)	N/A	N/A	6 (54.5)	N/A
No		4 (66.7)	2 (28.6)	1 (50)	N/A	N/A	5 (45.5)	
Grade	Mild	2 (33.3)	5 (71.4)	1 (50)	N/A	N/A	5 (45.5)	N/A
	Moderate	0 (0)	0 (0)	0 (0)	N/A	N/A	0 (0)	
	Severe	0 (0)	0 (0)	0 (0)	N/A	N/A	1 (9.1)	
Aortic regurgitation								
Yes		5 (83.3)	7 (100)	2 (100)	N/A	N/A	9 (81.8)	N/A
No		1 (16.7)	0 (0)	0 (0)	N/A	N/A	2 (18.2)	
Grade	Mild	2 (33.3)	2 (28.6)	1 (50)	N/A	N/A	6 (54.5)	N/A
	Moderate	3 (50)	5 (71.4)	1 (50)	N/A	N/A	3 (27.3)	
	Severe	0 (0)	0 (0)	0 (0)	N/A	N/A	0 (0)	

Categorical data are expressed as *n* (%). Phenotypes of aortic dilatation and grades of aortic stenosis or regurgitation are reported as percentages from the total count of cases among the specified subgroups; their summed counts and percentages correspond to the values noted with “Yes”. Statistical comparisons were performed using the Fisher’s exact test for categorical variables. Abbreviations: BAV, bicuspid aortic valve; 1RL, right–left cusp fusion; 1RN, right-non-coronary cusp fusion; 1LN, left-non-coronary cusp fusion; 2AP, anterior–posterior; 2LL, latero-lateral; N/A, not applicable.

**Table 4:** Comparison of blood biomarker profiles, aorta dimensions, and clinical features according to the presence of aortopathy in pediatric bicuspid aortic valve.

Parameter		Aortopathy		*p*-Value
		Yes (*n* = 13)	No (*n* = 13)	
Age, months		112 (26–179)	108 (69–169)	0.89
Gender, male		11 (84.6)	11 (84.6)	1 OR = 1 (CI 0.06–16.14)
MMP-2		198.2 (170.8–203.1)	192.9 (154.23–199.7)	0.65
MMP-9		345.9 (257.8–378.3)	365 (228.5–536.1)	1
TIMP-1		170.9 (159.6–181.9)	165.4 (149.7–181.9)	0.65
TGF-β1		107.5 (97.97–117.1)	100.6 (78.09–116.5)	0.36
MMP-9/TIMP-1		1.94 (1.57–2.75)	2.43 (1.28–3.99)	0.47
Annulus Ao	mm	17 (13–19)	17.5 (15–21)	0.62
Ao SV	mm	25.6 (20.12–28.25)	21 (20–23)	0.16
	Z score	1.20 (0.58–1.72)	0.48 (−1.2–1.23)	0.04
Ao STJ	Mm	20 (15.5–21)	23 (19.5–25)	0.41
	Z score	1.26 (1.06–2.19)	1.30 (0.71–1.5)	0.72
Asc Ao	Mm	25.5 (20–28)	19 (18–24)	0.06
	Z score	2.82 (2.45–3.17)	0.69 (0.46–0.87)	<0.05

Continuous data are median (25–75th percentile), unless otherwise stated; categorical data are *n* (%). Comparisons used Welch’s *t*-test or the Mann–Whitney U test for continuous variables and Fisher’s exact test for categorical variables. Biomarker concentrations are ng/mL. Annular values are raw diameters only; annular Z-scores were excluded because the diastolic annular measurement did not follow the Halifax systolic convention. Abbreviations: MMP, matrix metalloproteinase; TIMP, tissue inhibitor of metalloproteinase; TGF-β1, transforming growth factor-β1; Ao SV, sinuses of Valsalva; Ao STJ, sinotubular junction; Asc Ao, ascending aorta; OR, odds ratio; CI, confidence interval.

**Table 5:** Comparison of blood biomarker profiles, aorta dimensions, and clinical features according to aortic stenosis status in pediatric bicuspid aortic valve.

Parameter		Aortic Stenosis		*p*-Value
		Yes (*n* = 14)	No (*n* = 12)	
Age, months		103 (30.75–143.5)	156 (72.75–189.75)	0.10
Gender, male		12 (85.7)	10 (83.3)	1 OR = 1.19 (CI 0.07–19.27)
MMP-2		199 (172.47–213.22)	184.75 (147.2–199.17)	0.07
MMP-9		376.55 (291.9–561.62)	270.50 (209.4–397.75)	0.07
TIMP-1		173.25 (156.17–182.2)	163.25 (151.2–173.47)	0.39
TGF-β1		114.85 (102.37–120.8)	96.96 (85.87–105.55)	0.03
MMP-9/TIMP-1		2.44 (1.66–3.69)	1.73 (1.17–2.55)	0.32
Annulus Ao	mm	16.25 (13.5–17)	18.50 (16.87–21)	0.09
Ao SV	mm	21.5 (19–25.75)	25 (21–26.6)	0.16
	Z score	0.64 (0.39–1.29)	0.87 (−0.99–1.35)	0.88
Ao STJ	mm	19.75 (15.37–20.25)	25 (21.25–27)	0.10
	Z score	1.19 (0.88–2.01)	1.50 (1–2.33)	0.68
Asc Ao	mm	20.25 (18.12–27.37)	22.5 (18.75–27.12)	0.81
	Z score	2.63 (0.73–3.16)	1.22 (0.72–1.96)	0.15

Continuous data are median (25–75th percentile), unless otherwise stated; categorical data are *n* (%). Comparisons used Welch’s *t*-test or the Mann–Whitney U test for continuous variables and Fisher’s exact test for categorical variables. Biomarker concentrations are ng/mL. Annular values are raw diameters only; annular Z-scores were excluded. Abbreviations: MMP, matrix metalloproteinase; TIMP, tissue inhibitor of metalloproteinase; TGF-β1, transforming growth factor-β1; Ao SV, sinuses of Valsalva; Ao STJ, sinotubular junction; Asc Ao, ascending aorta; OR, odds ratio; CI, confidence interval.

**Table 6:** Comparison of blood biomarker profiles, aorta dimensions, and clinical features according to aortic regurgitation status in pediatric bicuspid aortic valve.

Parameter		Aortic Regurgitation		*p*-Value
		Yes (*n* = 23)	No (*n* = 3)	
Age, months		112 (64.5–173.5)	72 (49–132)	0.87
Gender, male		19 (82.6)	3 (100)	1 OR = 0 (0–15.39)
MMP-2		192.9 (156.26–199.8)	199.4 (170.2–205.55)	0.99
MMP-9		365 (245.75–540.65)	184.2 (163.5–233.7)	0.07
TIMP-1		169.6 (152.65–182.1)	166.9 (159.3–168.9)	0.69
TGF-β1		107.5 (96.18–117.45)	95.69 (94.11–100.29)	0.31
MMP-9/TIMP-1		2.13 (1.59–3.68)	1.10 (1.02–1.38)	0.05
Annulus Ao	Mm	17 (15–20.25)	17.5 (13.75–19)	0.83
Ao SV	Mm	22.5 (20–25.5)	28.6 (26.9–30.3)	0.12
	Z score	0.67 (−0.01–1.29)	1.77 (1.32–2.22)	0.27
Ao STJ	Mm	20 (15.75–22)	27 (27–27)	N/A
	Z score	1.26 (0.7–1.83)	4.24 (4.24–4.24)	N/A
Asc Ao	Mm	21 (18.25–27.25)	20.2 (18.6–24.1)	0.89
	Z score	1.35 (0.66–2.89)	2.26 (2.06–2.54)	0.54

Continuous data are median (25–75th percentile), unless otherwise stated; categorical data are *n* (%). Comparisons used Welch’s *t*-test or the Mann–Whitney U test for continuous variables and Fisher’s exact test for categorical variables. Biomarker concentrations are ng/mL. Annular values are raw diameters only; annular Z-scores were excluded. Abbreviations: MMP, matrix metalloproteinase; TIMP, tissue inhibitor of metalloproteinase; TGF-β1, transforming growth factor-β1; Ao SV, sinuses of Valsalva; Ao STJ, sinotubular junction; Asc Ao, ascending aorta; OR, odds ratio; CI, confidence interval; N/A, not applicable. No inferential Ao STJ comparison was performed because only one observation was available in the no-regurgitation subgroup.

**Table 7:** Association between aortic Z-scores and serum biomarker profile in pediatric bicuspid aortic valve subjects.

Biomarker	Ao SV Z-Score	Ao STJ Z-Score	Ascending Aorta Z-Score
**MMP-2**	ρ = 0.14; *p* = 0.49; *n* = 25	ρ = 0.52; *p* = 0.08; *n* = 12	ρ = 0.05; *p* = 0.79; *n* = 26
**MMP-9**	ρ = 0.12; *p* = 0.54; *n* = 25	ρ = 0.17; *p* = 0.58; *n* = 12	ρ = −0.01; *p* = 0.93; *n* = 26
**TIMP-1**	ρ = 0.22; *p* = 0.28; *n* = 25	ρ = −0.32; *p* = 0.29; *n* = 12	ρ = 0.02; *p* = 0.90; *n* = 26
**TGF-β1**	ρ = −0.02; *p* = 0.89; *n* = 25	ρ = −0.07; *p* = 0.81; *n* = 12	ρ = 0.06; *p* = 0.74; *n* = 26

Cells report Spearman’s ρ, nominal two-sided *p*-value, and complete-case *n*. Ao SV, sinuses of Valsalva; Ao STJ, sinotubular junction; MMP, matrix metalloproteinase; TIMP, tissue inhibitor of metalloproteinase; TGF-β1, transforming growth factor-β1; No multiplicity correction was applied.

**Table 8:** Association between aortic Z-scores and serum biomarker profile in pediatric tricuspid aortic valve subjects.

Biomarker	Ao SV Z-Score	Ao STJ Z-Score	Ascending Aorta Z-Score
**MMP-2**	ρ = −0.12; *p* = 0.62; *n* = 17	N/A (*n* = 1)	ρ = −0.05; *p* = 0.82; *n* = 17
**MMP-9**	ρ = 0.03; *p* = 0.89; *n* = 17	N/A (*n* = 1)	ρ = −0.08; *p* = 0.73; *n* = 17
**TIMP-1**	ρ = −0.26; *p* = 0.30; *n* = 17	N/A (*n* = 1)	ρ = −0.54; *p* = 0.02; *n* = 17
**TGF-β1**	ρ = 0.17; *p* = 0.51; *n* = 17	N/A (*n* = 1)	ρ = −0.15; *p* = 0.56; *n* = 17

Cells report Spearman’s ρ, nominal two-sided *p*-value, and complete-case *n*. Ao SV, sinuses of Valsalva; Ao STJ, sinotubular junction; MMP, matrix metalloproteinase; TIMP, tissue inhibitor of metalloproteinase; TGF-β1, transforming growth factor-β1; N/A, not estimable. No multiplicity correction was applied.

## Data Availability

The original contributions presented in this study are included in the article. Further inquiries can be directed to the corresponding authors.

## Abbreviations

Annulus Ao	aorta at the annulus
Ao STJ	aorta at the sino-tubular junction
Ao SV	aorta at the sinus of Valsalva
AR	aortic valve regurgitation
AS	aortic valve stenosis
Asc Ao	ascending aorta
BAV	bicuspid aortic valve
BMI	body mass index
BSA	body surface area
DBP	diastolic blood pressure
EF	ejection fraction
ELISA	Enzyme-Linked Immunosorbent Assay
FS	fractional shortening
HR	heart rate
IQR	interquartile range
LVEDV	left-ventricular end-diastolic volume
LVESV	left ventricle end-systolic volume
MMP-2	matrix metalloproteinase-2
MMP-9	matrix metalloproteinase-9
SBP	systolic blood pressure
SD	standard deviation
TAV	tricuspid aortic valve
TGF-β 1	transforming growth factor-β 1
TIMP-1	tissue inhibitor of metalloproteinase-1
WSS	wall shear stress
